# Nanopore sequencing technology and its applications

**DOI:** 10.1002/mco2.316

**Published:** 2023-07-10

**Authors:** Peijie Zheng, Chuntao Zhou, Yuemin Ding, Bin Liu, Liuyi Lu, Feng Zhu, Shiwei Duan

**Affiliations:** ^1^ Department of Clinical Medicine School of Medicine Zhejiang University City College Hangzhou China; ^2^ Institute of Translational Medicine, School of Medicine Zhejiang University City College Hangzhou China; ^3^ Key Laboratory of Novel Targets and Drug Study for Neural Repair of Zhejiang Province, School of Medicine Zhejiang University City College Hangzhou China

**Keywords:** nanopore sequencing, SARS‐CoV‐2, COVID‐19, cancer, plant, genome, mutation, pandemic

## Abstract

Since the development of Sanger sequencing in 1977, sequencing technology has played a pivotal role in molecular biology research by enabling the interpretation of biological genetic codes. Today, nanopore sequencing is one of the leading third‐generation sequencing technologies. With its long reads, portability, and low cost, nanopore sequencing is widely used in various scientific fields including epidemic prevention and control, disease diagnosis, and animal and plant breeding. Despite initial concerns about high error rates, continuous innovation in sequencing platforms and algorithm analysis technology has effectively addressed its accuracy. During the coronavirus disease (COVID‐19) pandemic, nanopore sequencing played a critical role in detecting the severe acute respiratory syndrome coronavirus‐2 virus genome and containing the pandemic. However, a lack of understanding of this technology may limit its popularization and application. Nanopore sequencing is poised to become the mainstream choice for preventing and controlling COVID‐19 and future epidemics while creating value in other fields such as oncology and botany. This work introduces the contributions of nanopore sequencing during the COVID‐19 pandemic to promote public understanding and its use in emerging outbreaks worldwide. We discuss its application in microbial detection, cancer genomes, and plant genomes and summarize strategies to improve its accuracy.

## Graphical abstract

Nanopore sequencing has been instrumental in managing the COVID‐19 pandemic. This review aims to increase public understanding of its role in identifying SARS‐CoV‐2 variants and tracking changes in regional epidemiology. Additionally, the review explores the use of nanopore sequencing in detecting other microbial outbreaks, as well as its applications in cancer and plant genomics.

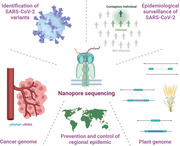

## 1 INTRODUCTION

The concept of nanopore sequencing, where single‐stranded nucleic acids pass through a nanopore in a membrane under an electric field, was first proposed by David Deamer in the 1980s.^1^ Despite initial skepticism, technological advances eventually made nanopore sequencing a reality.^2^ Proteins such as α‐hemolysin from Staphylococcus aureus^3^, ^4^, ^5^ and Mycobacterium smegmatis porin A (MspA)^6^, ^7^ were shown to distinguish the four bases on single‐stranded nucleotide molecules. The use of phi29 DNA polymerase slowed down the translocation of nucleic acid molecules through the nanopore, improving the signal‐to‐noise ratio.^8^, ^9^ Oxford Nanopore Technologies (ONT), founded in 2005 by Oxford professor Bayley and colleagues,^10^ facilitated the commercialization of nanopore sequencing with the release of their MinION sequencer in 2014.^2^

Currently, ONT has established a complete sequencing system process that includes advanced library preparation techniques combined with amplicon and other technologies, as well as numerous bioinformatics methods for analyzing and mining nanopore sequencing data.^2^

Since 2019, COVID‐19 caused by severe acute respiratory syndrome coronavirus (SARS‐CoV‐2) has spread to over 200 countries worldwide.^11^ As an RNA virus, SARS‐CoV‐2 continuously mutates during transmission, and the emergence of SARS‐CoV‐2 variants poses challenges for epidemic control.^12^ Obtaining a complete SARS‐CoV‐2 genome is crucial for detecting mutations because sequence changes can reduce the sensitivity of SARS‐CoV‐2 detection techniques.^13^ The first genome sequence of SARS‐CoV‐2 was obtained through metagenomic sequencing.^14^ Nanopore sequencing has played a significant role in the COVID‐19 pandemic.^15^ There is no doubt that nanopore sequencing has broad prospects, as demonstrated during the COVID‐19 pandemic. The portability, high efficiency, and low cost of nanopore sequencing make it particularly well suited for dynamically monitoring SARS‐CoV‐2 mutations and the spread of the COVID‐19 pandemic in different countries and regions. Currently, nanopore sequencing has been used for diagnostic sequencing of SARS‐CoV‐2,^16^ genome sequencing,^17^ and related research^18^ of SARS‐CoV‐2. In the field of pathogenic microorganism detection, nanopore sequencing has been used not only for detecting SARS‐CoV‐2 but also for identifying, typing, and monitoring the transmission of newly emerging monkeypox virus (MPXV)^19^ and norovirus (NoV).^20^ Nanopore sequencing can also be used to detect microbial communities in human tissues such as the skin and intestinal tract,^21^, ^22^ as well as in environmental samples.^23^ Its long single‐molecule long‐read sequencing capabilities make it widely used in cancer research^24^, ^25^ and clinical diagnosis and treatment.^26^, ^27^ Additionally, nanopore sequencing has unique value in resolving high‐quality plant genomes.^28^ However, public concerns about the error rate of nanopore sequencing still greatly limit its promotion and use. In reality, nanopore sequencing is a very cost‐effective technology,^29^ especially for underdeveloped regions. With the rapid iteration of ONT's sequencing platform and the development of new base‐calling algorithms, the accuracy of nanopore sequencing has greatly improved. Furthermore, its application during the COVID‐19 pandemic has proven that data obtained through nanopore sequencing is reliable.^30^

In this review, we have detailed the specific advantages of nanopore sequencing technology and its contributions during the COVID‐19 pandemic, including rapid identification of SARS‐CoV‐2, genotyping, dynamic transmission monitoring, and elucidation of related mechanisms of SARS‐CoV‐2. This work introduces the use of nanopore sequencing for detecting pathogenic microorganisms other than SARS‐CoV‐2, including viruses and bacteria. It also covers the application of nanopore sequencing in cancer research and clinical practice, as well as in plant genomics. Finally, we have analyzed the reasons for the low accuracy of nanopore sequencing and strategies for improving it. By introducing the contributions of nanopore sequencing during the COVID‐19 pandemic and its potential in other fields, we hope to promote the widespread application of next‐generation sequencing (NGS) technology and contribute to controlling new pandemics, including COVID‐19 or possible future ones.

## 2 MOLECULAR MECHANISMS OF NANOPORE SEQUENCING

### 2.1 Principle and specific process

**FIGURE 1:**
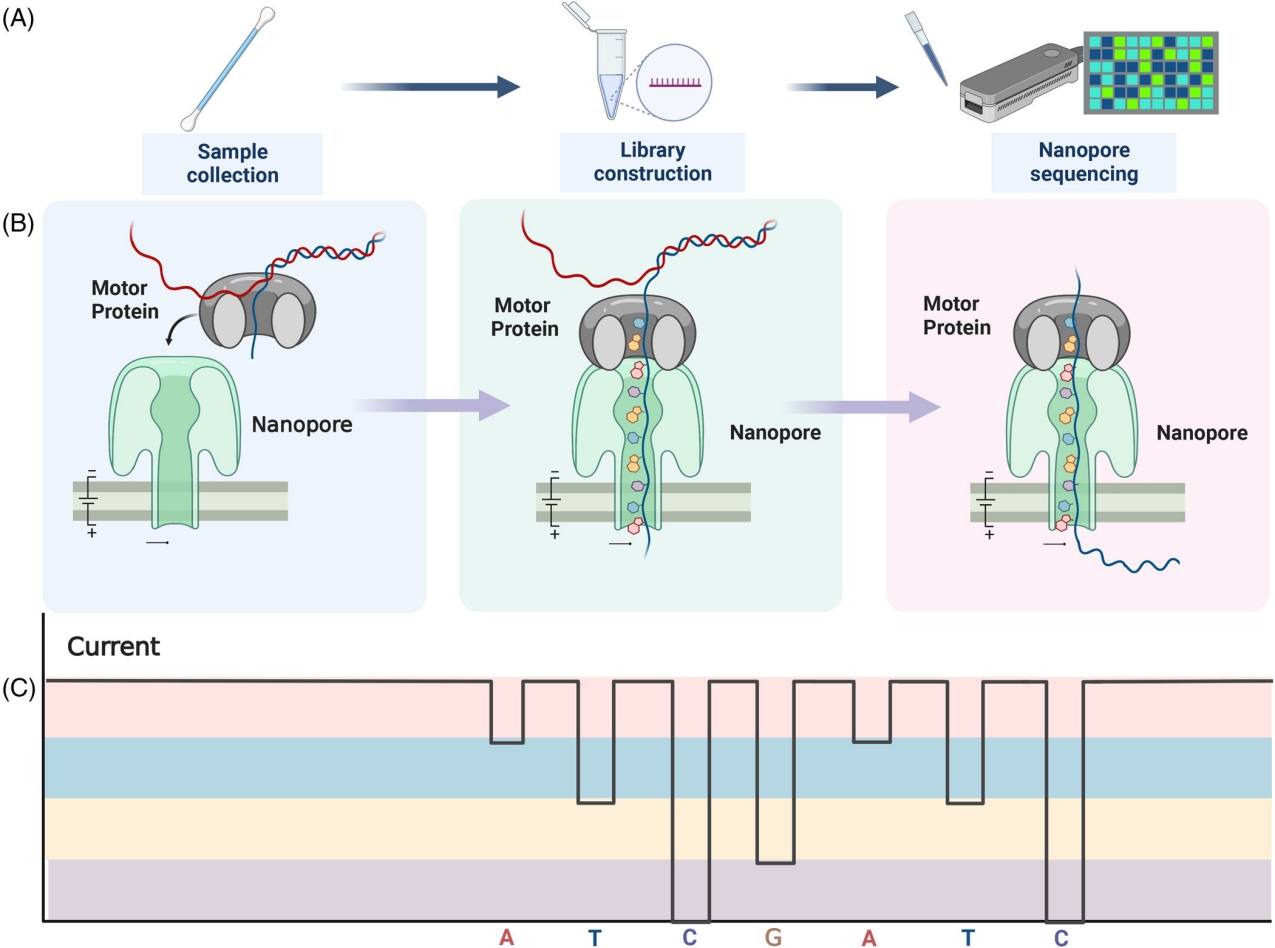
Schematic diagram of the basic principle and process of nanopore sequencing for SARS‐CoV‐2 detection. After the SARS‐CoV‐2 nucleic acid samples are collected, they can be added to the nanopore sequencer after simple library preparation, and the sequencing results can be obtained in real‐time (A). Motor proteins pull the nucleic acid through the nanopore, and the nucleic acid is detected and transmitted to the computer through the generated tiny current signal (B). The computer recognizes the base type by analyzing the current characteristic signal and converts the current signal into the base sequence (C).

During nanopore sequencing, nanopores act as biosensors.^1^ A certain number of nanopores are fixed on a resistive film, providing the only channel between both sides of the film. The ion solution on the cis side of the resistive film contacts the ion solution on the trans side through the nanopore.^1^ Electrodes are set at both ends of the nanopore sequencer to form a stable electric field. Nucleic acids (including DNA and RNA) will move to the nanopores under the action of an electric field in the nanopore sequencer.^31^, ^32^, ^33^, ^34^ The process of nucleic acid molecules passing through the nanopores is pulled by motor proteins, which can control the speed of nucleic acid passing through the nanopore.^2^ During the passage of nucleic acid through the nanopore, the charge of the nanopore changes, which in turn leads to a change in the flow of electrons on the resistive membrane. The electron flow caused by different bases or the quality of the modified molecular structure has unique characteristics.^2^ This electronic signal is captured and recorded by the nanopore sequencer, which is then passed through an algorithmic program to determine the base type and obtain the sequencing result^2^ (Figure 1).

### 2.2 Key components

The core of nanopore sequencing is the nanopore.^2^, ^35^ The nanopores used in initial experiments were derived from Staphylococcus aureus α‐hemolysin, a heptamer consisting of 14 antiparallel β‐strands with a diameter of approximately 2.6 nm.^36^ Other biological nanopores with similar activities can also be used for nanopore sequencing. For example, the protein Aerolysin, a pore‐forming toxin secreted by Aeromonas hydrophila,^37^ has a diameter of 1.0–1.7 nm and has been shown to enable sensitive single‐base–pair discrimination.^38^ The outer membrane protein G of Escherichia coli is also a potential candidate, but its open and closed conformational states make it currently unsuitable for single‐molecule sensing.^39^

MspA is currently the main choice for ONT's nanopore sequencing equipment. MspA is an octamer with a minimum inner diameter of 1 nm, which is narrower and more stable than the channel of α‐hemolysin, allowing it to have higher single‐nucleotide resolution.^40^ The difference in ionic currents of the four bases through the MspA nanopore is significantly greater than that of α‐hemolysin, allowing for more distinct distinction between the four bases.^41^ MspA also outperforms α‐hemolysin under extreme conditions, allowing it to adapt to harsh environments in emergencies.^42^

Motor proteins are critical for regulating the rate of nucleic acid translocation. Translocation of nucleic acids too fast will increase the difficulty of clip identification. In early experiments in the development of nanopore sequencing, single‐stranded DNA passed through the nanopore at a rate of 1–10 bases per millisecond, making it difficult to detect the sequencing signal.^10^ The unstable translocation rate also brings difficulties to subsequent data analysis. The phi29 DNA polymerase derived from bacteriophage was used as a molecular ratcheting system.^37^ Pairing MspA with phi29 DNAP slows DNA translocation for single‐base discrimination.^37^

### 2.3 Advantages and disadvantages brought by the sequencing mechanism

Nanopore sequencing's single‐molecule direct sequencing enables it to read ultra‐long DNA and RNA molecules in a timely manner, which cannot be achieved by first‐ and second‐generation sequencing. However, its disadvantages include a higher error rate (compared with previous generations of sequencing) and higher sample quality requirements. Despite this, nanopore sequencing is widely used in genome detection in fields such as microorganisms, tumors, animals and plants, especially during the COVID‐19 pandemic. Its advantages continue to emerge in various fields while its shortcomings become less obvious with continuous equipment updates, technology alliances, and analysis technology updates.

## 3 APPLICATION OF NANOPORE SEQUENCING TECHNOLOGY DURING THE COVID‐19 OUTBREAK

### 3.1 Detection of SARS‐CoV‐2 infection and the advantages of nanopore sequencing

#### 3.1.1 SARS‐CoV‐2 infection detection technology

SARS‐CoV‐2 tests can be classified into two types: nucleic acid‐based and protein‐based tests^43^ (Table 1). The primary methods for detecting SARS‐CoV‐2 nucleic acid are reverse transcription.

**TABLE 1:** Main detection methods of SARS‐CoV‐2.

Detection methods		Target	Advantages	Limitations	References
Molecular tests	RT‐PCR	Nucleic acid	The gold standard for virus detection; high sensitivity; suitable for large‐scale screening	Requires thermal cycling; prone to false negative results; time consuming	^44^, ^45^
	RT‐LAMP	Nucleic acid	Amplifies DNA under isothermal conditions; fast; high magnification; colorimetric measurement with strong visibility	Requires specific primers	^46^, ^47^
	Sequencing	Nucleic acid	High sensitivity; can specifically identify and differentiate a variety of coronaviruses and pathogenic microorganisms	Complicated operation; expensive	^48^
Serological tests	ELISA	Antibody	High sensitivity; qualitative or quantitative; ability to test multiple samples; low sample collection requirements	Not suitable for diagnosing acute infections; difficult to apply to mass screening	^49^, ^50^
	RDT	Antigen or antibody	Field test; simple and fast	Lack of high‐quality assessment; only qualitative analysis; questionable reliability of diagnostic results, use for research only.	^51^, ^52^
	Neutralization test	Antibody	Facilitate vaccine development	It needs to be carried out in a laboratory with a higher biosafety level	^53^

Current methods for detecting SARS‐CoV‐2 are mainly divided into two categories: nucleic acid‐based molecular tests and antigen‐ or antibody‐based serological tests. Molecular tests are divided into RT‐PCR, RT‐LAMP, and sequencing, and serological tests are divided into ELISA, RDT, and neutralization tests. RT‐PCR, reverse transcriptase‐polymerase chain reaction; RT‐LAMP, reverse transcription loop‐mediated isothermal amplification; ELISA, enzyme‐linked immunosorbent assay; RDT, rapid diagnostic test.

Polymerase chain reaction (RT‐PCR),^54^ reverse transcription loop‐mediated isothermal amplification (RT‐LAMP), and sequencing for diagnosis.^43^ RT‐PCR is the most commonly used method for detecting SARS‐CoV‐2 nucleic acid.^55^ It involves extracting viral RNA and converting it into complementary DNA (cDNA) using reverse transcriptase.^56^, ^57^ The cDNA is then used as a template to amplify the target gene using specific primers designed according to the SARS‐CoV‐2 genome and fluorescently labeled probes,^58^ allowing for the detection of SARS‐CoV‐2.^55^ While RT‐PCR is considered the “gold standard” for COVID‐19 diagnosis,^59^ it relies on the equipment and expertise of the testing center and the transportation time of samples can increase its time cost.^60^ Additionally, its higher false negative rate can pose challenges in managing the COVID‐19 pandemic.^43^ RT‐LAMP is an alternative technique that rapidly amplifies DNA through strand displacement by DNA polymerase after the initial synthesis of a dumbbell‐shaped DNA template.^61^ As a substitute for RT‐PCR,^60^ LAMP^62^ has low requirements for specialized equipment and provides fast diagnosis,^63^ making it one of the mainstream technologies for SARS‐CoV‐2 detection.^64^ However, its limited detection sensitivity restricts its widespread application.^65^ Sequencing for diagnosis involves obtaining the whole genome information of SARS‐CoV‐2 through metagenomic sequencing,^66^, ^67^, ^68^ including mainstream NGS Illumina and nanopore sequencing.^69^, ^70^ Its advantage is its high specificity and ability to simultaneously identify other respiratory viruses, compensating for the high false positives of RT‐PCR.^16^

Additionally, new molecular detection technologies are being developed or are expected to be used for SARS‐CoV‐2 nucleic acid detection. For instance, researchers have established a nucleic acid detection platform called specific high‐sensitivity enzyme reporter unlocking (SHERLOCK) by combining CRISPR–Cas enzymology with nucleic acid preamplification.^71^ This platform can be used for nucleic acid detection of Zika virus and dengue virus (DENV)^72^, ^73^ and also shows potential for SARS‐CoV‐2 nucleic acid detection.^74^, ^75^ Other technologies such as 3D‐printing^76^ and micro/nanorobots^77^ can also be used for SARS‐CoV‐2 detection. Researchers have created a disposable gene‐sensor using 3D pen‐printed electrodes (3D‐PP),^78^ where hybridization of ssDNA targeting the N gene of SARS‐CoV‐2 in the sensor to the SARS‐CoV‐2 RNA target affects the sensor signal.^79^ Plasmonic‐magnetic nanorobots^77^ are also available for SARS‐CoV‐2 RNA detection. This nanorobot is a plasmonic‐magnetic hybrid, composed of an Fe_3_O_4_ backbone and an outer surface of Au and Ag. When subjected to a transverse rotating magnetic field, it can actively flip and move with precise directionality. It is important to note that 3D printing^80^ and micro/nanorobots^81^ can also be utilized for the detection of SARS‐CoV‐2 antigens or antibodies. During the COVID‐19 pandemic, the global medical system has faced an unexpected crisis, prompting the application of 3D printing technology and the exploration of emerging technologies such as nanoscience in the medical field.^82^ This has proven to be both creative and promising. 3D printing technology has made significant contributions to the health system by producing personal protective equipment, sample collectors, safety accessories, and even isolation wards.^76^ Micro/nanorobots have advanced capabilities such as remote control, automatic propulsion, and precise positioning.^77^ However, in comparison with the nanopore sequencing for SARS‐CoV‐2 detection, the current applications of 3D printing and micro/nanorobots are limited and cannot obtain genome information of SARS‐CoV‐2.^83^, ^84^, ^85^ Additionally, cost considerations must be taken into account when compared with RT‐PCR for SARS‐CoV‐2 diagnosis. Nanopore sequencing can obtain SARS‐CoV‐2 genome information while detecting the virus and has been widely used during the COVID‐19 pandemic.^86^, ^87^, ^88^ Below, we will outline several advantages of nanopore sequencing for SARS‐CoV‐2 detection.

#### 3.1.2 Advantages of using nanopore sequencing for SARS‐CoV‐2 detection

Unlike RT‐PCR and RT‐LAMP, which are used for large‐scale population screening, sequencing has the advantage of obtaining the specific sequence of the virus strain. This is particularly useful during the widespread spread of SARS‐CoV‐2, where a large number of mutant strains have emerged and genome sequencing can aid in their identification and classification. Analyzing the similarity of the SARS‐CoV‐2 genome in different regions can also help track virus transmission. After the COVID‐19 pandemic began, researchers quickly identified this new coronavirus strain^14^ through metagenomic RNA sequencing on the Illumina platform. A genome‐wide comparison revealed that the SARS‐CoV‐2 genome was 96% identical to bat coronavirus, suggesting its possible origin.^89^ However, Illumina sequencing has drawbacks such as being time consuming, requiring complex data analysis, and being costly,^90^ limiting its application in some countries or regions, especially those that are economically and medically disadvantaged.^29^ These areas are also most likely to be affected by the COVID‐19 pandemic for a prolonged period and may produce more SARS‐CoV‐2 mutant strains.^91^ There is an urgent need to popularize sequencing technology in these areas to better understand the mutation,^92^, ^93^ transmission, and pathogenicity of SARS‐CoV‐2. Fortunately, next‐generation nanopore sequencing technology has effectively addressed the shortcomings of Illumina sequencing in terms of efficiency, convenience, and cost.^94^, ^95^, ^96^

Advantage 1: Real‐time and efficient. Nanopore sequencing can obtain the data required for sequencing in a few hours or less,^97^ making it more efficient than Illumina sequencing. This is mainly due to three advantages. First, the library preparation procedure for nanopore sequencing is simple. Direct sequencing eliminates the need for reverse transcription, amplification, and other processes required for Illumina sequencing, simplifying library construction and saving time while avoiding errors that may be introduced by redundant steps.^98^ When faced with low viral load, nanopore sequencing can also be combined with different amplification techniques to improve sequencing accuracy. Using a rapid barcoding kit, sublibrary construction based on amplification takes only ten minutes.^99^ Second, nanopore sequencing generates data in real‐time. Nucleic acid sequence information is collected as the nucleic acid passes through the nanopore.^97^ For COVID‐19 patient samples with high viral load (Ct 18–24), it takes only 1–2 h to generate data covering more than 90% of SARS‐CoV‐2.^99^ Finally, advanced bioinformatics analysis techniques enable real‐time data analysis,^2^ even for nonprofessionals.^100^

Advantage 2: Convenient and affordable. ONT's MinION device is a portable, field‐deployable device^99^ that facilitates the timely deployment of sequencing equipment after small‐scale outbreaks, particularly in economically disadvantaged communities that cannot afford to purchase large quantities of equipment. In terms of sequencing cost, the MinION device starts at only $1000.^101^ ONT is also committed to reducing the cost of nanopore sequencing. They have launched Flongle, an adapter for MinION that performs one‐time sequencing on small flow cells for only $100, making it a suitable option for community SARS‐CoV‐2 testing.^102^ A study comparing the cost of sequencing a single SARS‐CoV‐2 sample using the ONT and Illumina platforms found that the ONT platform costs only $10–40, significantly lower than the $150–250 required by the Illumina platform. This is partly due to the rapid barcoding library preparation method used by the ONT platform, which requires fewer reagents.^29^ Although the base error rate of nanopore sequencing samples is higher than that of Illumina sequencing,^29^ the high cost effectiveness of nanopore sequencing makes it worth promoting in economically disadvantaged areas.

Advantage 3: Long single‐molecule direct sequencing. Unlike second‐generation Illumina sequencing, nanopore sequencing has the unique advantage of long‐read direct sequencing of nucleic acid molecules.^90^ Its expanded use can advance our understanding of SARS‐CoV‐2 epigenetics and transcriptomics. Specifically, it has two advantages: direct sequencing and long‐read sequencing. Traditional sequencing requires reverse transcription and PCR amplification before sequencing the SARS‐CoV‐2 genome sequence,^90^ and additional specific chemical treatments are needed to capture RNA modifications for understanding SARS‐CoV‐2 epigenetics.^103^ These operations may introduce errors, but nanopore sequencing directly avoids these limitations. For instance, researchers have used nanopore sequencing to reveal the role of m6A RNA modifications in SARS‐CoV‐2 replication.^104^, ^105^ Additionally, the long‐read characteristic of nanopore sequencing means that its single‐stranded nucleic acid sequencing length is much longer than that of Illumina sequencing. In the face of the complex SARS‐CoV‐2 transcriptome, long‐read technology can easily obtain full‐length transcripts,^106^ allowing researchers to glimpse the subgenome landscape of SARS‐CoV‐2.^107^

#### 3.1.3 Nanopore sequencing is appropriate for detecting SARS‐CoV‐2

Low read accuracy is a well‐known limitation of nanopore sequencing, raising concerns about its accuracy in sequencing the SARS‐CoV‐2 genome.^108^ A study evaluating the effectiveness of nanopore sequencing for genomic analysis of SARS‐CoV‐2 used the ONT and Illumina platforms to sequence 157 samples. The researchers found that although the error rate of nanopore sequencing was higher than that of Illumina sequencing, it still achieved high‐precision determination of consensus‐level sequence and had a sensitivity and precision greater than 99% for monitoring single nucleotide variants (SNVs).^15^ Monitoring SNVs alone is sufficient for routine phylogenetic analysis,^30^ making nanopore sequencing an effective alternative to Illumina sequencing with trustworthy SARS‐CoV‐2 information. While short‐read sequencing led by Illumina sequencing remains the gold standard for SARS‐CoV‐2, the efficiency and cost advantages of emerging nanopore sequencing cannot be ignored, particularly in less developed countries or regions.

### 3.2 Library construction for nanopore sequencing for SARS‐CoV‐2 detection

**FIGURE 2:**
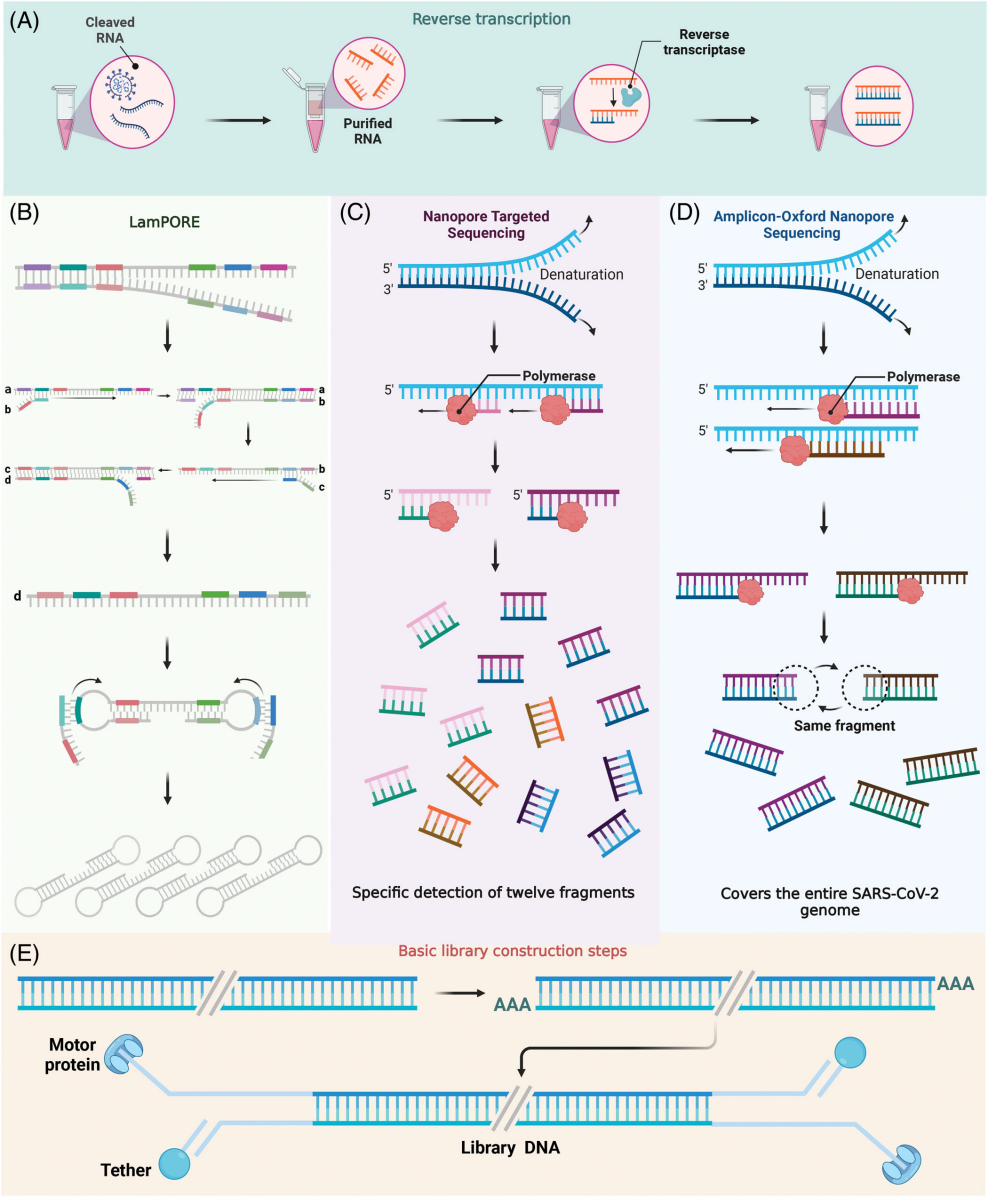
Nanopore sequencing library preparation and workflow for SARS‐CoV‐2 detection. Due to the low nucleic acid abundance of SARS‐CoV‐2 in samples, realistic library preparation for nanopore sequencing usually requires amplification of viral nucleic acids. The purified viral RNA needs to be reverse transcribed to obtain cDNA before amplification (A). Afterward, the researchers amplified the cDNA by LamPORE, NTS, and Amplicon‐Oxford nanopore sequencing. LamPORE technology uses LAMP technology to amplify cDNA (B). Both NTS and Amplicon‐Oxford nanopore sequencing use PCR technology to amplify cDNA. The difference is that NTS specifically amplifies 12 targets in the SARS‐CoV‐2 gene, which is more efficient and suitable for the detection of SARS‐CoV‐2 (C). Amplicon‐Oxford nanopore sequencing amplifies a large number of SARS‐CoV‐2 gene fragments, and it is beneficial to obtain the complete SARS‐CoV‐2 gene sequence by evaluating the overlap between the amplicons (D). After the above‐mentioned amplification is completed, the basic library construction process of nanopore sequencing is also required. First, add A bases to both ends of the nucleic acid to be tested to make the blunt ends become sticky ends, and then add Y linkers and motor protein. Among them, the motor protein is used to guide the nucleic acid into the nanopore, and there is also a tether on the Y linker, which acts as an anchor when the nucleic acid passes through the nanopore (E). LAMP, loop‐mediated isothermal amplification; NTS, nanopore targeted sequencing; PCR, polymerase chain reaction.

Unlike NGS technology, library preparation for nanopore sequencing does not require amplification,^109^ just adding motor proteins and adapters.^35^ In the detection of SARS‐CoV‐2, the abundance of viral nucleic acid is generally low,^110^ so the combination of different amplification techniques for nanopore sequencing is beneficial to improve the sensitivity of sequencing. A study evaluating the performance of different sequencing technologies found that at low viral loads, amplicon‐based enrichment methods were the most sensitive technology.^111^ The mainstream amplification method in nanopore sequencing is the same as the library construction method of next‐generation RNA sequencing, that is, reverse transcription of mRNA and amplify the double‐stranded cDNA obtained by reverse transcription, which is called Amplicon‐Oxford nanopore sequencing.^112^ In addition, nanopore targeted sequencing (NTS) is another amplification technology.^113^ Compared with amplicon‐based technology, NTS has higher detection efficiency and is more suitable for clinical detection of SARS‐CoV‐2.^16^ Loop‐mediated isothermal amplification (LAMP) is another amplification method that has certain advantages in combination with nanopore sequencing^114^ (Figure 2).

#### 3.2.1 Amplicon‐Oxford nanopore sequencing

In the case of low viral nucleic acid abundance, metagenomic sequencing methods require longer sequencing time and higher costs to analyze the sequence, and multiplex amplicons are a better choice for metagenomic sequencing library construction.^112^ Amplicon technology can help ONT sequencing obtain a high‐quality SARS‐CoV‐2 genome, compensating for the higher error rate of ONT sequencing compared with Illumina sequencing.^112^ Amplicon technology uses random primers to reverse‐transcribe purified RNA from patient samples to cDNA and performs multiple PCRs to generate the SARS‐CoV‐2 gene amplicon^115^ (Figure 2). The researchers used Amplicon‐Oxford nanopore sequencing to examine 42 samples, of which two out of the three the samples had 100% accurate sequencing results. In contrast, another one out of the three the samples had erroneous sequencing results due to poor sample quality and a concentration of purified amplicon less than 1 ng/μL. This highlights a limitation of nanopore sequencing compared with Illumina sequencing: it requires a higher input nucleic acid concentration for sequencing.^2^ In addition, increasing the length of the amplicon increases efficiency and reduces cost.^116^ Specifically, using the long amplicon protocol (∼2 and ∼2.5 kb) was preferred over the short amplicon protocol (∼400 bp).^117^

The ARTIC protocol is the most extensive amplicon‐based SARS‐CoV‐2 sequencing protocol.^118^ It relies on reverse transcription amplification of the SARS‐CoV‐2 genome using tiling and multiplexing amplicon protocols.^119^ Updates to this protocol (from version v1 to v4) address some of the primer set issues, such as primer aggregation, low coverage, and mutations in primer sites, and effectively reduce the cost of sequencing.^118^ At the same time, optimization can be carried out based on the ARTIC protocol (Table 2). For example, some researchers have designed two new primers to specifically amplify the immunodominant part of the spike protein gene. They then used S‐Protein‐Typer to automatically analyze mutations in the spike protein gene at the amino acid level. This method of specifically amplifying the immunodominant gene of the S protein increases sensitivity to mutations in the spike protein gene while reducing costs.^120^

**TABLE 2:** Amplicon‐based SARS‐CoV‐2 sequencing protocols.

Protocol		Public time	Optimized features	References
ARTIC protocol	ARTIC V1	2020.01.22	–	^125^
	ARTIC V2	–	Solving the problem of primers forming dimers	^125^
	Tiled‐ClickSeq	2020.03.11	Reduces generation of overlapping amplicons, increases sensitivity to RNA recombination, detects 5′‐UTR	^17^, ^121^
	ARTIC V3	2020.03.24	Increase coverage and reduce costs	^125^
	ARTIC V4	2020.06.18	Use variant sequences to solve the problem of amplicon loss and low genome sequence coverage caused by mutation of primer binding sites	^125^, ^126^
	S‐Protein‐Typer	2020.12	Identify S protein gene mutations	^127^
	"Midnight" 1200 bp amplicon split primer sets	2021.12.16	Streamline processes and increase efficiency	^122^
	–	2022.06.06	Improve efficiency	^119^
New protocol	CoronaHiT	2021.02.09	Flexible and high throughput	^123^
	Mini‐XT	2022.03.03	Low cost	^124^

Amplicon‐based SARS‐CoV‐2 sequencing protocols include updated versions of the mainstream ARTIC protocol, optimized versions based on the ARTIC protocol, and new protocols are being developed.

Compared with tiled amplicon technology, the ClickSeq method can detect 5'‐UTRs^17^, ^121^ that most previous tiled amplicon technology sequencing would miss. In addition, the 1200 bp amplicon split primer sets of the midnight method can shorten the sample processing time to one working day, which is valuable in small hospitals with small sample volumes.^122^ Additionally, using the ARTIC protocol with the Rapid Barcoding Sequencing kit reduces sample library preparation by 70 min.^119^

In addition, CoronaHiT and Mini‐XT are two new protocols for amplicon‐based sequencing of SARS‐CoV‐2.^123^, ^124^ Among them, the CoronaHiT protocol can provide the advantage of flexible high‐throughput,^123^ and Mini‐XT is a small library preparation solution. By using acoustic liquid transfer, Mini‐XT greatly reduces the use of reagents and other consumables while ensuring sequence quality, which reduces the cost of library preparation^124^ (Table 2).

#### 3.2.2 Nanopore targeted sequencing

Despite the significant progress made by nanopore sequencing over Illumina sequencing in terms of cost and efficiency, it remains more expensive and less efficient than RT‐PCR. As a result, NTS shifted its focus from obtaining the full genome sequence of SARS‐CoV‐2 to diagnosing and monitoring the virus.^16^ By using targeted sequencing on the nanopore sequencing platform, NTS was able to achieve high sequencing sensitivity while further reducing detection costs and time.^16^ NTS allows subsequent sequencing of amplified fragments specific to SARS‐CoV‐2 on the ONT platform^16^ (Figure 2). The specificity of NTS for SARS‐CoV‐2 reached 100% and compared with RT‐PCR, NTS had a higher recognition rate for positive infections.^16^ At the same time, NTS can effectively recognize mutant nucleic acid sequences, which is beneficial to classify SARS‐CoV‐2 variants.^16^ By adding primers for other respiratory viruses, NTS can not only detect SARS‐CoV‐2 infections but also simultaneously monitor more than 10 other respiratory viruses, including influenza A and B viruses and parainfluenza virus.^16^ This allows for timely identification of patients with coinfections of SARS‐CoV‐2 and other respiratory viruses. As the global COVID‐19 pandemic continues^128^ and emerging epidemics such as MPXV^129^ threaten to place additional strain on medical systems in underdeveloped areas,^130^ NTS offers a solution to overcome the high false negative rates of traditional RT‐PCR while also monitoring for coinfections. This improves diagnostic efficiency and reduces the risk of cross‐infection.

#### 3.2.3 LamPORE

LamPORE is a new platform that combines LAMP technology with nanopore sequencing.^114^ Specifically, LamPORE is a process for reverse transcription of purified RNA to generate cDNA, followed by LAMP amplification and subsequent sequencing on the nanopore sequencing platform^114^ (Figure 2). Nanopore sequencing can improve the low sensitivity of LAMP technology, making LamPORE technology consistent with RT‐PCR in sensitivity.^114^ Both prospective and retrospective studies have confirmed the accuracy of the LamPORE technology for the detection of SARS‐CoV‐2.^131^ At the same time, LAMP technology brings the possibility of high‐throughput detection to nanopore sequencing.^114^ One LamPORE device can complete the detection of 480 samples in 1 h.^132^ In addition, LamPORE can be used to detect SARS‐CoV‐2 variants.^132^

### 3.3 Bioinformatics tools to analyze nanopore sequencing data of SARS‐CoV‐2

Nanopore sequencing produces a unique FAST5 data format and its long‐read, direct sequencing features are not available in traditional short‐read sequencing. As a result, bioinformatics tools developed for traditional short‐read sequencing cannot meet the analysis requirements of nanopore sequencing. After the release of ONT's commercial sequencing equipment, researchers developed numerous analysis tools based on specific analysis needs. Following the outbreak of the COVID‐19 pandemic, many analytical tools suitable for SARS‐CoV‐2 monitoring were also developed. Their functions mainly focus on monitoring SARS‐CoV‐2 mutations or integrating operations to simplify and improve interactivity. In this section, we highlight newly developed bioinformatics analysis tools for these outbreaks (Table 3)

**TABLE 3:** Bioinformatics analysis of nanopore sequencing.

Category	Analysis method	Download URL	Function	References
Variant detection	VirPool	Using a probabilistic model to estimate the proportion of SARS‐CoV‐2 mutations present in wastewater	–	^133^
	Variabel	Accurate identification of intrahost variants with low allele frequencies	www.gitlab.com/treangenlab/variabel	^134^, ^135^
	–	Process FASTQ reads into spike gene consensus sequences to accurately call spike protein variants.	–	^136^
	S‐Protein‐Typer	Identification of SARS‐CoV‐2 variants by monitoring mutations in the spike protein gene	https://github.com/MassimoGregorioTotaro/s‐protein‐typer	^120^
	LDV‐Caller	Identifying SARS‐CoV‐2 variations from low‐depth sequencing data at reduced costs	–	^137^
Simplified integration	ONTdeCIPHER	The process involves integrating data to reconstruct the consensus genome for each viral isolate. Variants and their effects are then identified, and the lineage is inferred. Finally, multisequence alignments and phylogenetic analyses are performed.	https://github.com/emiracherif/ONTdeCIPHER	^137^
	MALVIRUS	Integrated, easy to install, easy to use for SARS‐CoV‐2 genotyping	https://algolab.github.io/MALVIRUS	^138^
	CalmBelt	Integrated and interactive interface for tracking virus spread and spotting potential spreading clusters	https://github.com/BioML‐CM/CalmBelt	^139^
	poreCov	Integration for genotyping and genome reconstruction of SARS‐CoV‐2	https://github.com/replikation/poreCov	^140^
	EDGE COVID‐19	This workflow uses an integrated and intuitive web‐based interface to automate data quality control, reference‐based genome variant and consensus calling for SARS‐CoV‐2, and lineage determination.	https://edge‐covid19.edgebioinformatics.org https://github.com/LANL‐Bioinformatics/EDGE/tree/SARS‐CoV2	^141^
	NanoCoV19	Integrated, efficient, surveillance, and lineage analysis for SARS‐CoV‐2	‐	^142^
Others	BugSplit	Sensitive and specific identification of SARS‐CoV‐2 through high‐precision taxonomic binning of metagenomic data	https://bugseq.com/academic	^143^
	ReadItAndKeep	This fast and lightweight tool scans sequence data and retains only the data that maps to the viral genome. This prevents data leakage related to the human genome.	https://github.com/GenomePathogenAnalysisService/read‐it‐and‐keep	^144^
	LeTRS	By identifying leader‐TRS junctions, the abundance of sgmRNAs can be quantitatively measured to study the biological characteristics of SARS‐CoV‐2.	https://scicrunch.org/resolver/RRID:SCR_022138	^145^
	Genopo	A smartphone can be used for portable offline analysis of DNA methylation in human genome samples.	https://mobilegenomics.ce.pdn.ac.lk/genopo/download.html	^100^
	InterARTIC	Simple interactive interface to help ordinary users reconstruct consensus genome sequences.	https://github.com/Psy‐Fer/interARTIC/	^146^
	Prediction	Determine optimal sequencing run times, reduce costs, and improve data quality.	–	^147^
	AccuVIR	Genome assembly	–	^148^

During the COVID‐19 pandemic, several bioinformatics methods have been proposed to optimize the results of nanopore sequencing of SARS‐CoV‐2. The application of deep learning or model prediction has reduced the difficulty of data analysis and, consequently, the cost of sequencing.

#### 3.3.1 Application 1: variant detection

Due to higher error rates in nanopore sequencing, some low‐occurrence mutations may be masked.^135^ This is important to consider when studying within‐host variation of SARS‐CoV‐2, as it may affect how hosts respond to treatment.^149^ To address this issue, researchers have developed a variant call filtering tool called Variabel. This tool compares samples from different patients or from the same patient at different times to distinguish between true variants and sequencing errors.^135^ An evaluation of patient‐derived datasets from multiple viruses, including SARS‐CoV‐2, showed that Variabel can accurately identify mutations with an allele frequency below 0.5.^134^, ^135^ Additionally, deep learning can also improve SARS‐CoV‐2 variant detection in ONT sequencing. Researchers trained a generative adversarial network called LDV‐Caller to analyze low‐depth sequencing results at a level comparable to high‐depth sequencing, greatly reducing the cost of whole‐genome sequencing.^137^ Detecting new variants of SARS‐CoV‐2 is also important. The spike protein is key for SARS‐CoV‐2 to bind to host cells,^150^ and a large number of mutations in the spike protein can lead to immune escape.^135^ Nanopore sequencing can detect mutations in the spike protein gene individually, rather than using traditional whole‐genome sequencing.^136^ This can be done by amplifying the gene of the immunodominant part of the S protein using PCR and analyzing it with tools like S‐Protein‐Typer.^120^ Additionally, VirPool can estimate the proportion of SARS‐CoV‐2 variants in wastewater samples by analyzing nanopore sequencing data using probabilistic models.^133^

#### 3.3.2 Application 2: simplified integration

Analyzing ONT sequencing results requires a certain level of bioinformatics knowledge, which can limit its widespread use.^146^ To address this, several interactive applications have been developed for free use on mobile phones^100^ and computers.^140^, ^141^, ^146^ These applications use intuitive graphical interfaces, workflows, and job scheduling systems to improve interactivity and make bioinformatics analysis techniques more accessible.^139^, ^146^, ^151^ This allows even users without any bioinformatics background to analyze data from SARS‐CoV‐2 sequencing.^146^ Initially, these tools were designed for rapid analysis of the complete genome sequence of SARS‐CoV‐2.^100^, ^140^, ^146^ Now they can also perform rapid typing, lineage analysis, and phylogenetic analysis of SARS‐CoV‐2 by integrating established nomenclature assignments, GISAID clades, and PANGO lineages.^139^, ^142^, ^151^ This helps regional public health systems respond quickly to COVID‐19 outbreaks..

### 3.4 Nanopore sequencing for analysis of SARS‐CoV‐2 epidemiological information

The ongoing COVID‐19 pandemic has had a significant socioeconomic impact worldwide. However, it has also promoted the use of sequencing technology to detect virus mutations, track the virus's origin and dynamic evolution, and predict epidemic transmission and transmission chains.^152^ The large amount of SARS‐CoV‐2 genomic data generated in a short period of time has greatly contributed to the analysis of the virus's epidemiological information.^152^ Nanopore sequencing has been widely used during the pandemic, with rapid whole‐genome sequencing and analysis contributing to public health decision‐making and early assessment of SARS‐CoV‐2 transmission.^153^ Specifically, nanopore sequencing technology contributes to the prevention and control of the COVID‐19 pandemic in three main ways.^154^ First, it can quickly obtain genome sequence information from SARS‐CoV‐2 samples,^155^ allowing for timely monitoring and understanding of the invasion of foreign SARS‐CoV‐2 lineages and the diversity of SARS‐CoV‐2 genomes in a region.^156^ Second, long‐term monitoring of SARS‐CoV‐2 lineages in the same area can help understand the dynamic replacement of lineages in that region. Different regions may have different mutation lineages of SARS‐CoV‐2 due to varying selection pressures,^157^ facilitating exploration of the underlying mechanisms of SARS‐CoV‐2 evolution by understanding spatial heterogeneity during the pandemic.^158^ Third, nanopore sequencing can be used to trace the origin and spread of SARS‐CoV‐2, including interregional and intracommunity transmission. Phylogenetic analysis of the sequenced SARS‐CoV‐2 genome and known SARS‐CoV‐2 sequence information from other regions can help determine the most likely source of infection in newly^159^ emerged areas,^160^ providing a reliable reference for rapid public decision‐making.^161^ Additionally, nanopore sequencing can also help identify cases of SARS‐CoV‐2 reinfection.^162^

#### 3.4.1 Identification of SARS‐CoV‐2 variants

The results of nanopore sequencing are beneficial to understand the diversity of the SARS‐CoV‐2 genome within a specific region. Timely identification and sequencing of emerging SARS‐CoV‐2 mutations is beneficial for monitoring the evolutionary direction of the virus and predicting changes in viral infectivity and virulence.^163^ The identification of emerging VOCs in areas where the virus is rapidly progressing can help control the spread of the outbreak.^164^

The application of nanopore sequencing identified a large number of emerging SARS‐CoV‐2 mutations (Table 4), and also specifically identified some VOCs with high transmissibility and high virulence.^164^ In addition, the identification of SARS‐CoV‐2 variants by nanopore sequencing can help understand the susceptibility of populations to different SARS‐CoV‐2 variants after vaccination.^165^

**TABLE 4:** Nanopore sequencing for identifying SARS‐CoV‐2 mutations.

Region	Time	Lineage	Nucleotide changes	Amino acid changes/transcriptional changes	Samples	References
Hangzhou, China	2020.1–2020.3	–	33 substitutions	21 amino acid variants in S, N, ORF1a/b, and ORF3a	29	^166^
Tuscany, Italy	2020.4	B1.1	5 single nucleotide changes (at positions 241, 3037, 144,08, 19,839, and 23,403) and mutations of 3 consecutive nucleotides (GGG→AAC) at position 28881	D614G mutation	1	^167^
10 different districts, India	2020.3–2020.6	A2a, A4, and B	–	38 amino acid substitutions, include 24 in ORF1ab, 5 in S protein, 2 in ORF 3a, 1 each in E, M, and ORF 7a, and 4 in N protein	26	^168^
Nanjing, China	2020–2021	B.1.1.7, B.1.351, P.1, and B.1.617.2	328 nucleotide mutation sites include 205 nonsynonymous mutations and 112 synonymous mutations	ORF1ab, S, ORF3a, and N	41	^169^
France	2021.1	B.1.640	46 nucleotide substitutions and 37 deletions	30 amino acid substitutions and 12 deletions	13	^170^
Mumbai, India	2020.8–2020.9, 2021.5–2021.10	B.1.1.306, B.1.36, B.1.1.32, B.1.1.281, B.1.617.2, B.1.617.2, K417N, AY.120, AY.38, and AY.99	642 mutations	D616G mutation, spike(S) mutation, nucleocapsid mutation	155	^171^, ^172^
Colombia	2021.1–2021.4	B.1.621	–	I95I, Y144T, and Y145S	–	^173^
Bangladesh	2021.6–2021.7	B.1.617.2 and B.1.1.529	Spike (S) gene deletion	Spike (S) mutation, T19R, L452R, T478K, P681R, and D950N	52	^174^, ^175^
Colorado, America	2021.6	AY.1, AY.2, and AY.3	ORF1b:V2354F and ORF7a:Q94* S112L	NSP15:V303F and Premature stop codon	34	^176^
Arkansa, America	–	–	T265I, A3529V, G18C, Q57H, S201G, R203K, and G204R	D615G mutation	2	^177^

Nanopore sequencing can quickly obtain the specific gene sequence of SARS‐CoV‐2 and rapidly identify emerging mutant strains. By detecting nucleotide changes in mutant strains, possible viral amino acid changes can be inferred.

Currently, nanopore sequencing is used in several regions for the identification of SARS‐CoV‐2 VOCs.^178^ During the first wave of SARS‐CoV‐2 in the UK, researchers tested 563 SARS‐CoV‐2 samples from Oxford using nanopore sequencing. They detected 479 SARS‐CoV‐2 genetic variants, more than half of which involved changes in the amino acid sequence of the encoded protein of the viral genome.^179^ In addition, they found that the number of genetic variants of SARS‐CoV‐2 was significantly increased in summer samples compared with spring samples.^179^ Following a localized outbreak of SARS‐CoV‐2 infection at a Canadian hospital, researchers performed nanopore sequencing on samples from the hospital. The results showed that mutations in the spike protein gene were associated with headache symptoms in patients.^180^, ^181^ In southern France, nanopore sequencing identified a novel SARS‐CoV‐2 variant, B.1.640.2, in which the spike protein appeared with substitutions of N501Y and E484K.^170^ In Colombia, nanopore sequencing identified the B.1.621 lineage involving amino acid substitutions in the spike protein Y144T, Y145S, R346K, and so on, resulting in B.1.621 being a VOCs.^173^ The researchers developed a protocol based on nanopore sequencing in Paraguay based on Spike protein variants, a RT‐PCR for detecting Spike receptor binding domain, which was developed to rapidly identify SARS‐CoV‐2 VOCs during the Paraguay outbreak.^99^, ^182^ After PCR identified the first four omicron variants in Germany, nanopore sequencing was used for rapid sequencing to confirm the PCR results.^183^ In addition, nanopore sequencing also identified lineages such as B.1.1.7, B.1.1.318 in the Republic of Gabon^184^ and lineages such as B.1.351 and P.1 in Nice, France.^185^ Nanopore sequencing identified a large number of SARS‐CoV‐2 mutant sequences. Including identifying 25 sublineages of SARS‐CoV‐2 B.1.1.529 in Bangladesh between December 2021 and January 2022.^174^, ^186^ SARS‐CoV‐2 B.1.617.2 sublineages were identified in 12 children in Chittagong, Bangladesh from June 2021 to July 2021.^175^

In addition to VOCs, nanopore sequencing also helped to discover variants of SARS‐CoV‐2 in different regions. For example, five SARS‐CoV‐2 variants^171^ were identified in Mumbai, India, between August and September 2020. Eight SARS‐CoV‐2 variants were found in rural United States^177^ and Mexico.^187^ SARS‐CoV‐2 Siena‐1/2020 lineage and Cali‐01 lineage were discovered in Italy^167^ and Colombia,^188^ respectively, in March 2020. Also, SARS‐CoV‐2 genomes were sequenced from March–April 2020 in Hong Kong^189^ and August–September 2020 in Malta.^190^

#### 3.4.2 Tracking regional SARS‐CoV‐2 lineage changes

The turnover of the SARS‐CoV‐2 lineage within the region may have resulted from viral evolution^168^ or the continuous introduction of foreign dominant mutants.^191^ Sequencing samples at different times in the region is beneficial to understand the dynamic replacement process of the SARS‐CoV‐2 lineage in the region^168^ (Table 5).

**TABLE 5:** SARS‐CoV‐2 lineage replacement and transmission routes in the analysis area using nanopore sequencing.

Regional lineage replacement	Region	Time	Original lineage (%)	Replaced lineage (%)	Samples	References
	Ribeirao Preto City, Brazil	2020.3–2020.4/2021	B1.1.33 (61.1%), B.1.1 (27.8%), and B.1.1.28 (11.1%)	P.1 (91%) and P.2 (9%)	29	^192^
	Sierra Leone	2020.10/2021.6	Second wave, R.1	Third wave, B.1.617.2	65	^193^
	Indonesia	2021.1/2021.7	First wave, B.1.466.2	Second wave, AY.23, AY.24, AY.39, AY.42, AY.43, and AY	202	^194^
	Congo	2020.3–2020.8/2021.2–2021.3/20216–2021.9	The first and second waves, B.1.214.2	Third wave, Delta (B.1.617.2)	74	^191^

Interregional spread	Region	Time	Lineage	Speculated source	Samples	References
	North‐Rhine Westphalia, Germany	2020.2–2020.3	–	Netherlands, China	55	^195^
	Ecuador	2020.3–2021.1	HEE‐01 (B)	Netherlands	4	^196^
			HGSQ‐USFQ‐018 (B1)	United Kingdom		
			HGSQ‐ USFQ‐007 HGSQ‐USFQ‐010 (B)	Scotland		
	India	2020.3	A2a	Europe and America	104	^197^
			A3	Middle East		
			A4	Southeast Asia (Indonesia, Thailand, and Malaysia), and Central Asia (Kyrgyzstan)		
	Tunis	2020.3	B.1	Morocco, Ghana, Uganda, and the Democratic Republic of Congo (DRC)	2	^127^
			A	China		
	Hangzhou, China	2020.1–2020.3	–	–	29	^166^
	Baltimore‐Washington metropolitan area	2020.3	–	–	114	^198^
	Cape Town, South Africa	2020.3–2020.10	B.1.1.29 B.1.8	Europe	50	^199^
	Netherlands	2020.3	–	Germany	14	^200^
	Islamabad, Pakistan	2020.11–2021.2	B.1.1.250	United Kingdom Bangladesh	4	^201^
		2020.11–2021.2	B.1.261	Saudi Arabia	4	^201^
	Ukraine	2021.7	Delta	Central and Eastern European countries	24	^202^
	Sierra Leone	2020.3–2021.8	B.1.617.2 lineage (Delta variant)	England, USA, and Scotland	130	^193^
	Italian Province of Trento	2020.3–2021.12	B.1 B.1.1.29	Lombardy	253	^203^
	Nanjing, China	2020–2021	B.1.1.7	Italy, Philippines, and Argentina	42	^169^
			B.1.351	Philippines		
			P.1	Argentina		
			B.1.617.2	Russia and England		
	Pakistan	2020.12–2021.2	B.1.1.7 B.1.36 B.1.1.212	United Kingdom	35	^204^
	Armenia	2020–2022	B.1.1.7	Iran, Italy, New Zealand, Russia, Jordan, Germany, and India	145	^205^

Obtaining the specific gene sequence of SARS‐CoV‐2 by nanopore sequencing is beneficial to analyze the epidemiological information of SARS‐CoV‐2. Including understanding the lineage replacement of different waves of SARS‐CoV‐2 epidemics in the region, and inferring the origin of the SARS‐CoV‐2 lineage in the region through phylogenetic analysis.

Nanopore sequencing results reveal SARS‐CoV‐2 evolutionary trends in central India, with the highly infectious spike protein D614G variant continuing to replace other lineages.^168^ The Delta lineage predominated in SARS‐CoV‐2 infection in Indonesia.^194^ A reduction in some SARS‐CoV‐2 lineages was associated with vaccination status in the Mumbai region of India.^172^ In Sierra Leone,^206^ the second and third waves of SARS‐CoV‐2 infection consisted of different lineages. Of these, the second wave was the R.1 lineage and the third was the B.1.617.2 lineage, which resulted from continued foreign introductions.^193^ In the Democratic Republic of Congo, nanopore sequencing identified seven SARS‐CoV‐2 lineages in three waves and observed 289 variants, and phylogenetic analysis revealed multiple introductions of SARS‐CoV‐2 lineages from different origins.^191^ Two waves of outbreaks in the Brazilian city of Ribeirao Preto were analyzed using nanopore sequencing. The results showed that the B1.1.33 lineage was the main lineage in the early stage of the epidemic, and the B.1.1 and B.1.1.28 lineages were also identified; while the second wave of the epidemic was dominated by the P.1 lineage, in addition to the P.2 lineage.^192^ In the Karachi region of Pakistan, the A222V variant in the second wave coexisted with the highly contagious D614G variant in the first wave.^207^

#### 3.4.3 Mapping SARS‐CoV‐2 transmission

After nanopore sequencing of SARS‐CoV‐2 in the new outbreak area, phylogenetic analysis was performed between the SARS‐CoV‐2 data obtained by sequencing and the virus sequence information in the known region, and the origin of the new outbreak can be inferred.^198^ At the same time, nanopore sequencing was also used to detect the SARS‐CoV‐2 outbreak in the community, including the early invasion of SARS‐CoV‐2,^208^ the speed of transmission,^158^ and the target of transmission.^209^

*Monitoring the spread of SARS‐CoV‐2 between regions*: Sequencing the SARS‐CoV‐2 genomic lineage by nanopore technology is beneficial to understand the introduction events of foreign SARS‐CoV‐2 and the transmission route within the region (Table 5).

Sample sequences from Hangzhou, China, from January to March 2020, suggest multiple overseas origins of SARS‐CoV‐2.^166^ Multiple foreign sources of the 2020–2021 outbreak in Nanjing, China were analyzed by nanopore sequencing, including Argentina, Italy, the Philippines, Russia, and the United Kingdom.^169^ The nanopore sequencing results of the SARS‐CoV‐2 strain from the outbreak in Heinsberg, Germany, from February to March 2020, showed that it was only related to the SARS‐CoV‐2 strain from the Netherlands.^195^ Subsequently, sequencing results of infection cases in the Dutch town of Sittard in early March 2020 showed that the outbreak may have originated in Heinsberg,^200^ Germany, which borders Sittard. Nanopore sequencing of SARS‐CoV‐2 isolates from Ecuador, Africa, from the March 2020 outbreak to January 2021 revealed three distinct origins, including the Netherlands, the United Kingdom, and Scotland.^196^, ^210^ Nanopore sequencing of the SARS‐CoV‐2 genome in India revealed that the outbreak originated from the introduction from Europe, the United States, and Asia after March 2020.^197^ More than a quarter of the samples in Maryland and Washington, DC, between March 11 and 31, 2020, were analyzed by nanopore sequencing. The results indicated that the outbreak came from multiple introductions at different locations worldwide.^198^ Phylogenetic analysis of sample sequences from the South African outbreak from March to October 2020 revealed that at least nine independent introductions occurred early in the South African outbreak.^199^ From March 2020 to August 2021, the SARS‐CoV‐2 lineage in Sierra Leone, Africa, showed that it may have originated from England, the United States, and Scotland.^193^ From March 2020 to December 2020, an analysis of the viral genome from the province of Trentino in Italy showed that it was from the Lombardy region.^203^ From November 2020 to February 2021, nanopore sequencing of the SARS‐CoV‐2 lineage in the surrounding area of Pakistan showed that the outbreak originated from the introduction of the United Kingdom and Bangladesh.^201^ The July 2021 outbreak of the SARS‐CoV‐2 Delta variant in Ukraine mainly came from Middle Eastern and Eastern European countries.^202^ Nanopore sequencing of SARS‐CoV‐2 strains in the Karachi region of Pakistan revealed that their outbreaks from December 2020 to February 2021 were related to the UK SARS‐CoV‐2 strain.^204^ The 2020–2022 virus lineage in Armenia shows that it may have originated in Iran, Italy, New Zealand, Russia, Jordan, Germany, India, and so on.^205^

Nanopore sequencing can be used to monitor urban wastewater and predict the spread of VOCs across a country.^211^ In Italy, researchers collected 332 wastewater samples from 20 regions and autonomous provinces between October and November 2022 and sequenced them using both Sanger and nanopore sequencing.^211^ They found that the proportion of samples containing mutations from the BQ.1 and BQ1.1 lineages increased significantly in November, reaching 43%.^211^ As expected, the variant that was prevalent before being replaced by BQ.1/BQ.1.1 in late 2022 quickly became the dominant SARS‐CoV‐2 lineage in Italy.^211^

By further analyzing existing sequencing data and studying the general patterns and differences of SARS‐CoV‐2 genomes in different regions, researchers can gain a better understanding of the virus's evolution and transmission modes.^212^ One study analyzed 186,682 samples of SARS‐CoV‐2 isolates from different locations worldwide and classified them into 303 subgroups using PhenoGraph, an unsupervised learning classifier.^212^ The origin of these subgroups was then inferred based on their distribution in different countries and regions.^212^ Another study analyzed 444,478 SARS‐CoV‐2 genome data and identified 42 comutation modules, which refer to simultaneous mutations that occur when the virus mutates under environmental selection pressure.^213^ The sample sources were then grouped based on these comutation modules to determine the phylogenetic relationship between groups.^213^ The widespread use of sequencing technology worldwide is therefore helpful in inferring the origin and dynamic spread of the epidemic. The affordability and portability of nanopore sequencing will facilitate the adoption of sequencing technology, enriching the universality and extensiveness of sequencing data in databases.

Monitoring community transmission of SARS‐CoV‐2 through nanopore sequencing, researchers can understand the SARS‐CoV‐2 lineage in the community and observe the transmission process of SARS‐CoV‐2 in the community.^214^ Nanopore sequencing can be used to detect SARS‐CoV‐2 gene fragments in social waste water, which facilitates early detection of SARS‐CoV‐2 variant invasion.^208^ After the emergence of new variants of SARS‐CoV‐2, the researchers sampled sewage in the community and found several typical VOCs including Alpha, Delta,^164^ and Omicron.^215^ In Düsseldorf, Germany, nanopore sequencing results indicated a potential link between SARS‐CoV‐2 outbreaks in local populations and hospital outbreaks.^216^ Using nanopore sequencing results, the researchers calculated that the doubling time of the SARS‐CoV‐2 BA.2 in dwellings was only 1.28 days.^158^ The study by Bosnia and Herzegovina revealed the phenomenon of SARS‐CoV‐2 transmission between humans and pets.^209^ The nanopore sequencing results showed that pets in the home of the confirmed patient were also infected with the same SARS‐CoV‐2 as the owner.^209^ In a recent long‐term study, researchers monitored the concentration of SARS‐CoV‐2 RNA in wastewater from a university compound over a period of 2.5 years. Using nanopore sequencing, they were able to detect the genome of the Omicron variant strain in the samples.^217^

#### 3.4.4 Identification of SARS‐CoV‐2 reinfection

A rare case of SARS‐CoV‐2 reinfection has emerged in Colombia, with two infections identified by nanopore sequencing as lineages B.1 and B.1.1.269.^218^ By analyzing the variation of the SARS‐CoV‐2 genome in the GISAID database, the researchers speculated that this patient had multiple SARS‐CoV‐2 variants.^219^ They then sequenced 42 COVID‐19 patients using nanopore sequencing and ultimately found that nearly half of them had SARS‐CoV‐2 variant coinfection.^219^ In addition, some COVID‐19 patients who had recovered and been discharged tested positive again for the virus using RT‐PCR, even though they were no longer carrying it. Nanopore sequencing revealed that these patients had highly degraded SARS‐CoV‐2 genomes, representing approximately 34.5% of the full genome. Researchers believe that these re‐positive cases may be due to the shedding of cells containing residual SARS‐CoV‐2 nucleic acid in the patient's body. This suggests that these recovered patients had very low infectivity, particularly through respiratory transmission.^162^

### 3.5 SARS‐CoV‐2 infection and other infections

Metagenome sequencing provides the composition of all microorganisms in a sample.^220^ Metagenome sequencing was performed in patient samples, enabling direct detection of SARS‐CoV‐2.^221^, ^222^ By employing nanopore sequencing technology for metagenomic analysis, we can obtain complete influenza virus sequence information directly from respiratory samples taken from patients. This provides the possibility to distinguish SARS‐CoV‐2 infection from other infections and to diagnose SARS‐CoV‐2 infection with other infections.^221^

Oropharyngeal commensal microorganisms seem to have a certain impact on the severity of symptoms after host infection with SARS‐CoV‐2.^223^ By using nanopore sequencing to analyze the full‐length 16S rRNA gene, researchers found that the oropharyngeal flora of patients infected with SARS‐CoV‐2 was dysregulated. Opportunistic pathogens such as Peptostreptococcus anaerobius and Pseudomonas stutzeri were found to be enriched in these patients.^224^ This may eventually lead to coinfection in patients with COVID‐19, whose mycobacterial and mycoplasma enrichment in the nasopharynx is closely associated with subsequent chest pain and fever.^225^ Notably, a recent study found that SARS‐CoV‐2 coinfection with L. mirabilis was common in asymptomatic COVID‐19 cases.^226^

The influx of critically ill patients into intensive care units due to COVID‐19 has led to a sharp increase in personnel density. These patients often require invasive treatments or examinations, such as invasive ventilation, and are at risk of secondary infections. Nanopore sequencing can quickly identify and type these infections, aiding in the diagnosis and treatment of COVID‐19 patients with secondary infections.^227^ Real‐time whole‐genome sequencing on the ONT platform can also monitor persistent infections and assess changes in drug resistance after treatment, providing guidance for medical staff to make appropriate treatment decisions for individual patients.^228^ It is worth noting that the results of nanopore sequencing also revealed the risk of secondary infection with a drug‐resistant bacterial infection in COVID‐19 patients, in which a hospital in New York City found secondary infection of carbapenemase‐producing Enterobacterales (CPE) in the COVID‐19 outbreak.^229^ Nanopore sequencing was used to rapidly genotype CPE, detect antibiotic resistance genes, and perform phylogenetic analysis in this infection.^229^ Notably, clinical metagenomics using nanopore sequencing can identify pathogens and predict bacterial resistance in just 8 h, allowing for timely antibacterial treatment for patients.^227^

Middle East respiratory syndrome coronavirus (MERS‐CoV) is a zoonotic disease that has been prevalent in the Middle East since 2012. MERS‐CoV and SARS‐CoV‐2 are both coronaviruses and patients may be coinfected with both viruses.^230^ In the Middle East, a large number of MERS‐CoV‐infected patients have similar symptoms to SARS‐CoV‐2‐infected patients.^231^ The combination of nanopore sequencing and amplicon technology helped to rapidly differentiate between the two infections.^231^ By combining nanopore sequencing with multiplex isothermal amplification, SARS‐CoV‐2 can be differentiated from a variety of viruses including influenza A virus (IAV), and human adenovirus.^232^ By combining nanopore sequencing with RT‐PCR technology, the researchers were able to simultaneously screen 41 viruses including MERS‐CoV^233^ while identifying SARS‐CoV‐2.

### 3.6 Nanopore sequencing to study the molecular mechanism of SARS‐CoV‐2 pathogenicity

#### 3.6.1 Direct sequencing of the SARS‐CoV‐2 transcriptome and epitranscriptome

When using nanopore sequencing for clinical diagnosis of SARS‐CoV‐2 infection or monitoring mutations, amplification techniques are often used in conjunction with sequencing due to the generally low virus titer in clinical samples. This helps reduce the error rate of sequencing and the complexity of follow‐up analysis. Research into the specific pathogenic mechanism of SARS‐CoV‐2 can better demonstrate the advantages of direct sequencing and long‐reads provided by nanopore sequencing.

**FIGURE 3:**
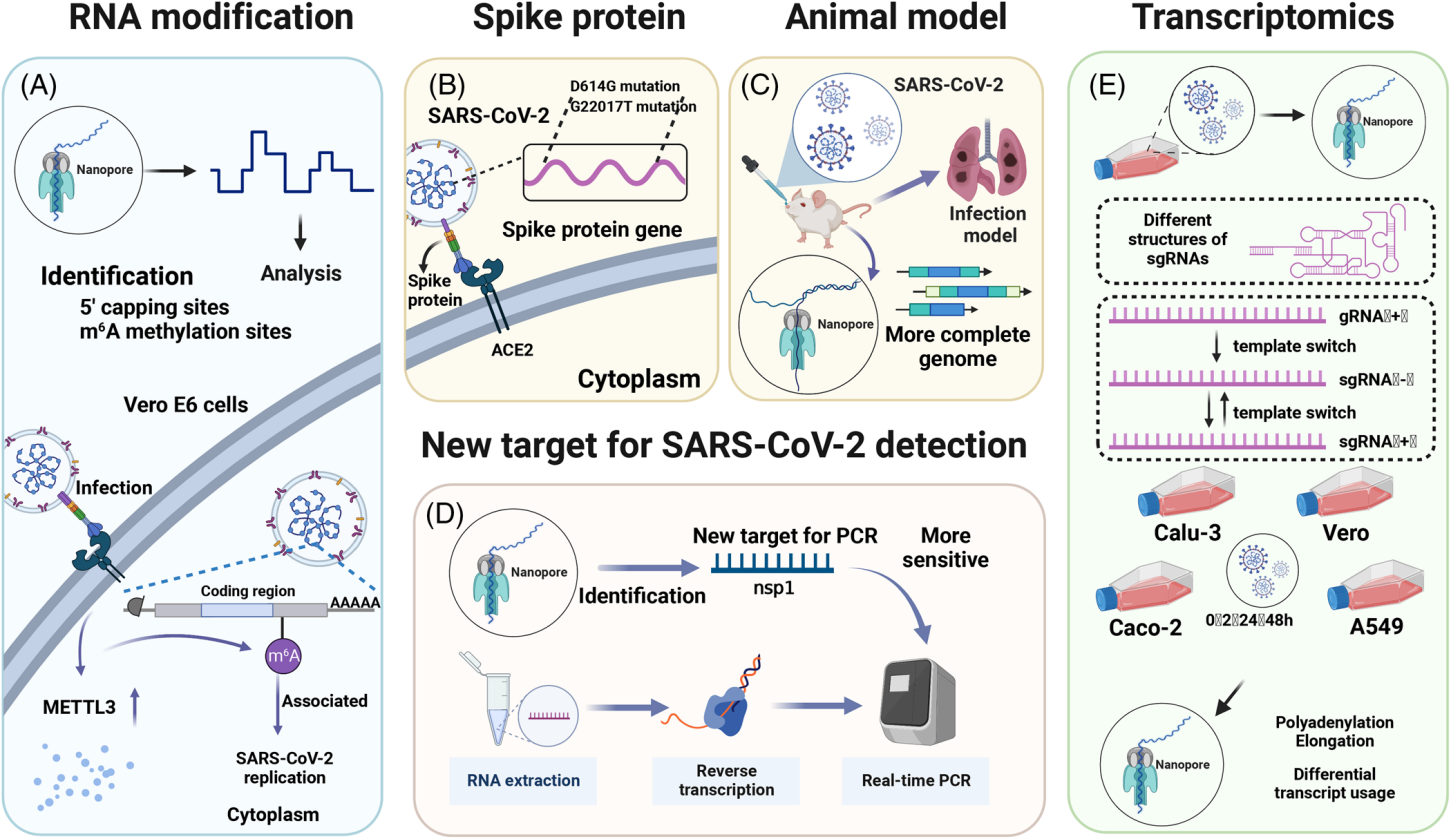
Nanopore sequencing was used to study the pathological mechanism of SARS‐CoV‐2. Nanopore sequencing advances the understanding of RNA modifications and transcriptomics of SARS‐CoV‐2. In the study of RNA modification, nanopore sequencing identified 5′ capping sites as well as m6A methylation sites in the SARS‐CoV‐2 genome. The m6A modification has been implicated in SARS‐CoV‐2 replication, and the production of m6A modification requires METTL3, which is upregulated after SARS‐CoV‐2 infection (A). In the study of transcriptomics, through nanopore sequencing of Vero cells infected with SARS‐CoV‐2, the researchers learned about the different structures of sgRNAs and the template switch process in sgRNA production, which involves the production of positive and negative sgRNAs (E). In addition, nanopore sequencing facilitates the study of mutations in the spike protein gene involved in host cell recognition by SARS‐CoV‐2 (B). The long‐read feature of nanopore sequencing has also helped to obtain the complete genome of an animal model of SARS‐CoV‐2 infection (C). Nanopore sequencing identifies the nsp1 gene as a novel target for RT‐PCR detection of SARS‐CoV‐2 (D). METTL3, methyltransferase‐like 3; m6A, N6‐methyladenosine; nsp1, nonstructural protein 1; sgRNAs, subgenomic RNAs.

*Transcriptomic studies of SARS‐CoV‐2*: Studying the SARS‐CoV‐2 transcriptome can reveal specific components of the virus's genome and clarify novel events in its transcriptional process. This can help us understand the pathogenic mechanism of SARS‐CoV‐2 and provide new possibilities for developing clinical tools for prevention, diagnosis, and treatment.^107^ While Illumina sequencing has higher accuracy,^18^ it is limited by its short reads. Nanopore sequencing, on the other hand, can perform long‐read direct sequencing, which is especially useful for capturing full‐length transcripts and identifying novel transcripts not recognized by short reads.^234^ By combining nanopore and Illumina sequencing, researchers have uncovered the complex transcriptomic architecture of SARS‐CoV‐2, including subgenomic RNAs (sgRNAs), open reading frames (ORFs), and transcription‐regulatory sequences (TRSs). They also discovered that SARS‐CoV‐2 differs from other coronaviruses in its unconventional RNA joining events, which deviate from the classical TRS‐mediated mechanism^107^ (Figure 3).

Nanopore sequencing has also revealed regulatory signatures of the SARS‐CoV‐2 subgenome.^107^, ^235^ Specifically, SARS‐CoV‐2 sgRNAs can form different structures.^235^ and are generated through successive template switches that rely on RNA interactions.^107^ Template switching can occur in both directions, including during positive‐strand synthesis.^107^ Nanopore sequencing has provided insight into the role of the transcription complex in SARS‐CoV‐2 template switching^236^ (Figure 3).

When replicating in vivo, SARS‐CoV‐2 may be forced to delete certain regions from its genome, which could affect its infectivity or responsiveness to vaccines.^237^, ^238^ Transcriptome analysis using nanopore sequencing has revealed the absence of a furin‐like cleavage site‐encoding portion in the transcriptome of the SARS‐CoV‐2 spike protein.^135^ This cleavage site is used to split the spike protein into functional subunits, increasing the virus's infectivity.^239^ One study reported its deletion in over half of the transcripts encoding the spike protein.^237^

Host cells respond differently to SARS‐CoV‐2 infection.^240^ Researchers sequenced infected and mock‐infected human lung adenocarcinoma cells (Calu‐3), human colon cancer cells (Caco‐2), and African green monkey kidney epithelial cells (Vero) using the ONT platform. They found that GSDMB and KPNA2 gene transcripts^240^ were associated with SARS‐CoV‐2 infection in different cells. ONT sequencing also confirmed that SARS‐CoV‐2 did not integrate into the DNA of infected HEK293T cells^241^ (Figure 3).

*Studies on RNA modification in SARS‐CoV‐2*: One benefit of nanopore direct sequencing is its ability to accurately identify epigenetic modifications on bases. As nucleic acid molecules pass through the nanopore, any modifications on the base will affect the nanopore's surface current. By recording changes in the current in real‐time, it is possible to identify RNA modifications of SARS‐CoV‐2 after analysis.^242^ RNA sequencing results of SARS‐CoV‐2 using the ONT platform have revealed its epitranscriptomic landscape.^18^ The results showed that the SARS‐CoV‐2 transcript has at least 41 RNA modification sites, including N6‐methyladenosine (m6A), 5‐methylcytosine methylation (5mC), and 2′‐O‐methylation (Nm). These modification sites most frequently occur on the AAGAA sequence.^18^ Additionally, modified RNA has a shorter poly(A) tail than unmodified RNA.^18^ Studies have also found that SARS‐CoV‐2 genomic RNA has more modifications at multiple sites compared with sgRNA,^243^ and that sgRNA modifications remain stable during the infection process^243^ (Figure 3). The abundance of epigenetic modifications in SARS‐CoV‐2 may be related to maintaining the stability of its viral RNA.

m6A modification is the most prevalent type of RNA modification and requires methyltransferase‐like 3 (METTL3) for its production.^244^ After infection with SARS‐CoV‐2, Vero E6 cells exhibit increased expression of METTL3 and its translocation from the nucleus to the cytoplasm.^105^ Using a method called mixed‐weight neural bagging (MWNB), nanopore sequencing has been able to identify m6A modifications with a high accuracy of 97.85%.^245^ ONT sequencing results have shown that SARS‐CoV‐2 RNA contains m6A modifications and that removing these modifications inhibits the replication of SARS‐CoV‐2.^105^ This provides an effective target for treating SARS‐CoV‐2 infections.^104^ Additionally, an increase in m6A modifications in the SARS‐CoV‐2 gene has been found to be associated with immune evasion by SARS‐CoV‐2^246^ (Figure 3).

Nanopore ReCappable Sequencing (NRCeq) is a new technique based on the ONT platform that can identify full‐length sgRNA of SARS‐CoV‐2.^247^ Its strength lies in its ability to distinguish intact full‐length sgRNAs from degraded sgRNA fragments.^248^ The 5’ end of sgRNA has a specific m7G cap structure, and full‐length sgRNA can be recognized by monitoring this cap.^249^ NRCeq can identify full‐length sgRNA of SARS‐CoV‐2 and map its 5’‐capping site by replacing m7G caps with azido‐modified caps.^106^, ^247^

#### 3.6.2 Development of a novel detection method for SARS‐CoV‐2

RT‐PCR is the most widely used molecular detection technology for monitoring SARS‐CoV‐2. However, the frequent mutations of the virus present a significant challenge for primer design.^250^ When a mutation occurs in the SARS‐CoV‐2 sequence targeted by RT‐PCR primers, the primers may no longer effectively amplify the target sequence, resulting in decreased detection sensitivity. Nanopore sequencing identified a nonstructural protein 1 (nsp1) gene in the SARS‐CoV‐2 genome.^251^ This gene was highly expressed in samples from individuals infected with SARS‐CoV‐2 of varying severity.^251^ The researchers developed a new RT‐PCR assay targeting the nsp1 gene and validated it using 101 clinical samples, with a sensitivity of 93.1% and a specificity of 100%. This achieved the detection sensitivity of conventional RT‐PCR^251^ (Figure 3).

#### 3.6.3 Mutation detection of spike protein

The infection process of SARS‐CoV‐2 involves the binding of the spike protein to the host cell angiotensin‐converting enzyme 2 receptor.^150^, ^252^ The spike protein gene has been a key target for RT‐PCR detection of SARS‐CoV‐2.^253^ The study of the spike protein is an important part of the mechanism study of SARS‐CoV‐2. Samples from the South American country from March to April 2020 were sequenced using ONT platform and showed that the D614G substitution in the spike protein gene is common in South America.^254^ Another study sequenced samples from January to March 2020 using ONT platform and found the G22017T variant, which corresponds to the W152L mutation of the spike protein^255^ (Figure 3).

#### 3.6.4 Sequencing animal models of SARS‐CoV‐2

The Syrian hamster (Mesocricetus auratus) is an important animal model in SARS‐CoV‐2 research because SARS‐CoV‐2 isolates can cause severe lung damage in Syrian hamster lungs. The imaging characteristics of this lung injury resemble those seen in patients with pneumonia following infection with SARS‐CoV‐2.^256^ Benefiting from the advantages of long‐read nanopore sequencing, the application of nanopore sequencing allowed researchers to generate higher‐quality Syrian hamster genome sequences, which would be more beneficial for analyzing host responses to SARS‐CoV‐2 infection^257^ (Figure 3). Advancing nanopore sequencing technology for reference genomes of animal models such as macaques, ferrets, and cats^258^ will aid in understanding the relationship between virus pathogenicity and genetic differences across species.

## 4 APPLICATIONS OF NANOPORE SEQUENCING IN OTHER MICROBIAL DETECTION

Nanopore sequencing has played an undeniable role in the COVID‐19 pandemic. However, its application in microbial detection extends beyond SARS‐CoV‐2 and COVID‐19 prevention and control. It has been used to detect the genomes of human infectious viruses such as seasonal influenza,^259^ MPXV c, Zika,^260^ and dengue,^261^ as well as animal and plant viruses such as African swine fever virus,^262^ Lily virus,^263^ and Potato virus Y (PVY).^264^ Nanopore sequencing is also used to detect bacteria through 16S rRNA gene sequencing or whole‐genome sequencing to analyze microbial communities in the intestinal tract,^21^ nasal cavity,^265^ and skin^22^ of normal individuals or patients. It can also confirm the specific typing of patient‐infected strains and detect drug resistance‐associated gene mutations.^266^ Additionally, nanopore sequencing has been applied to the detection of foodborne pathogens through the detection of specific genes or whole‐genome sequencing to identify pathogen types and predict their potential virulence characteristics.^267^

### 4.1 Virus detection

#### 4.1.1 Detection of viruses in clinical samples

Nanopore sequencing's advantages in clinical virus genome detection lie in its affordability, portability, efficiency, and long‐read capabilities. Its affordability and portability make it easy to popularize and promote, particularly in countries or regions with limited medical resources. This facilitates the timely identification of emerging pathogen types and helps prevent and control epidemics. Its ability to efficiently obtain complete viral genome data is also useful for detecting new virus mutants or tracking the spread of the virus. During the COVID‐19 pandemic, nanopore sequencing played an important role in preventing and controlling regional epidemics caused by other viruses. For example, in Indonesia, where over 100,000 people are infected with DENV annually, researchers used the portable nanopore MinION platform to sequence amplified DENV, improving epidemiological surveillance in resource‐poor settings.^261^ Similarly, in Central Africa, where MPXV poses a threat to public health, one study evaluated real‐time sequencing of the MPXV genome using the MinION sequencer to better understand its transmission. The results showed that the sequencing data generated by nanopore sequencing were complete and sufficient for phylogenetic analysis, providing accurate information about the virus source.^268^

Influenza viruses can cause seasonal epidemics that pose significant challenges to human health and public health systems. Accurate detection is crucial for patient management and epidemic control. A UK study reported the use of nanopore sequencing for influenza surveillance in a clinical setting, where researchers performed metagenomic sequencing on 180 patient samples. The results showed an 83% sensitivity and 93% specificity for detecting IAV.^268^ Nanopore sequencing was also used to identify clonotypic diversity in the IAV genome and, when combined with Illumina sequencing, increased the accuracy of clonotype identification to 98.2%.^269^ Frequent mutations in IAV may reduce RT‐PCR detection of gene targets, but nanopore sequencing can identify new diagnostic targets for IAV mutants.^270^ By sequencing clinically infected patients, researchers identified PB2 and NS genes as new IAV diagnostic targets and found that using them for RT‐PCR detection had lower detection limits than traditional M genes.^270^

#### 4.1.2 Animal and plant virus detection

Plant and animal viruses can cause significant economic losses in agriculture and animal husbandry worldwide.^271^ Symptoms of plant viral infections are often atypical and nonspecific. While traditional sequencing technology has been widely used for detecting and identifying plant viruses, it is not suitable for on‐site detection and can be costly.^272^ Nanopore sequencing's portability and flexible throughput make it uniquely adaptable for plant virus detection.^271^ It has been used to detect viruses in common economic crops such as potato, wheat, broad bean,^273^ lily,^274^ and jasmine.^275^ For example, nanopore sequencing can distinguish between virus infection and nutrient deficiency in early‐stage wheat growth and accurately identify Wheat streak mosaic virus while revealing variations in its resistance gene Wsm2.^276^ It is also used for genotyping PVY, with whole‐genome sequencing of different PVY types showing over 95% consistency with the consensus sequence obtained by Illumina sequencing.^264^

Animal virus infections can spread rapidly and have severe socioeconomic consequences. Nanopore sequencing has been used to identify various animal viruses, including differentiating between three Capripox Viruses in outbreaks in the Middle East and Africa,^277^ identifying African horse sickness in an outbreak in Thailand,^278^ and tracking Enzootic Bovine Leukosis caused by Bovine leukemia virus transmission in US pastures.^279^ It has also been used to reveal the complex transcriptional landscape of porcine reproductive and respiratory syndrome virus.^280^

### 4.2 Detection of microbial populations

By using nanopore sequencing for metagenomic or 16S rRNA sequencing, researchers can analyze the diversity of microbial populations in the environment or human tissues. The portable and real‐time MinION sequencing system released by ONT is suitable for field biological exploration to study microbial communities and has been used in various locations, including the Antarctic,^23^ Arctic,^23^ tropical desert^281^ regions, and even the International Space Station. Its resilience to harsh environments^282^ and ability to provide power and data transfer over a single USB 3.0 connection^283^ enabled real‐time sequencing on the International Space Station, reducing transportation costs and avoiding sample degradation during storage.^283^

In a healthy physiological state, certain types and quantities of microorganisms live on the human body surface and body cavity. Imbalances in microbiota are linked to the development of various diseases.^284^, ^285^ While short‐read 16S rRNA sequencing has commonly been used to analyze microbial populations in humans, full‐length 16S rRNA sequencing using nanopore sequencing technology is an effective and low‐cost alternative.^286^, ^287^ It has been used for sequencing the endometrial,^287^ skin,^22^ and gut^286^ microbiomes.

Metagenomic sequencing can reveal the specific composition and function of bacterial, viral, and eukaryotic mixtures in the microbiome. While traditional short‐read sequencing produces incomplete and fragmented microbial genomes,^21^ long‐read nanopore sequencing can assemble contiguous bacterial isolate genomes.^21^ Nanopore sequencing can also detect DNA methylation in bacteria and microbiomes. Researchers developed a method called nanodisco to identify reliable DNA methylation^288^ when applied to individual bacteria or the small intestinal microbiome of mice.

## 5 APPLICATION OF NANOPORE SEQUENCING IN CANCER GENOMES

**FIGURE 4:**
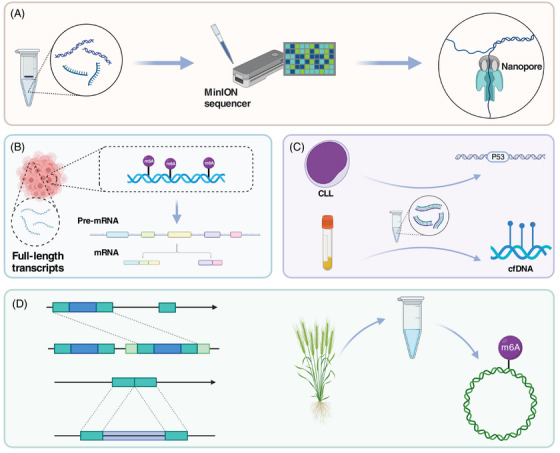
Nanopore sequencing for the detection of cancer and plant genomes. The exceptional performance of nanopore sequencing's single‐molecule long‐reads has made it a popular choice for sequencing cancer and plant genomes (A). In cancer research, nanopore sequencing is used to obtain transcripts and epigenetic modifications, as well as to understand the process of AS (B). Clinically, it can be used for gene analysis and liquid biopsy in patients (C). In plant research, nanopore sequencing is used to analyze and obtain high‐quality genomes (D). cfDNA, cell‐free DNA; CLL, chronic lymphocytic leukemia; m6A, N6‐methyladenosine.

Cancer is characterized by genomic changes and its development is accompanied by progressive changes in the genome and epigenome.^289^, ^290^ Sequencing technology has deepened our understanding of cancer genome changes and the emergence of NGS technology and high‐performance computers has enabled comprehensive analysis of the cancer genome at both basic research and clinical levels.^289^ Sequencing technology is widely used in basic cancer genome research and its deepening has promoted clinical applications such as personalized cancer therapies through molecular target detection or cancer biomarker screening.^291^ Nanopore sequencing's advantages in cancer research lie in its ability to perform direct single‐molecule long‐read sequencing, capturing cancer transcriptome^24^, ^25^ and epigenetic changes.^292^ Its long‐read capabilities make it more sensitive to structural variations (SVs) in cancer genomes^293^, ^294^ and better able to identify alternative splicing sites in gene expression.^295^, ^296^ In clinical applications, nanopore sequencing's portability and speed make it a useful tool for biopsy,^297^ particularly liquid biopsy.^297^ Its long‐read capabilities can capture special DNA fragments in blood that cannot be achieved by short‐read sequencing^298^ (Figure 4).

### 5.1 Basic research on cancer

While RNA direct sequencing is possible on traditional sequencing platforms,^299^ most RNA‐Seq experiments, including those for cancer transcriptomics, are performed on instruments that sequence converted DNA molecules.^300^ This requires the preparation of RNA into cDNA.^300^ Similarly, detection of epigenetic modifications is also indirect.^301^ For example, DNA 5mC modification is detected through Bisulfite sequencing (BS‐Seq) and high‐throughput sequencing of m6A, the most common modification in eukaryotic mRNA, relies on m6A‐specific antibodies.^302^, ^303^ Nanopore sequencing can sequence RNA without PCR amplification and directly detect different types of epigenetic modifications,^301^ avoiding complex sample handling procedures or potential bias from reverse transcription. Its long‐read capabilities help identify full‐length tumor transcripts, AS cleavage sites, and accurately locate SVs in the cancer genome.^25^ One study used nanopore sequencing to directly sequence the transcriptomes of tumors and adjacent tissue from three hepatocellular carcinoma (HCC) patients and found 361 novel transcripts significantly associated with HCC patient prognosis. Another study analyzed the transcriptomes of 42 pairs of HCC and matched noncancerous liver samples using nanopore sequencing and detected 46,663 transcripts, one‐tenth of which were novel discoveries.^304^ Nanopore sequencing revealed AS events in early stages of HCC carcinogenesis.^304^ Another study used nanopore sequencing to perform whole‐genome sequencing of 11 HCC samples from Japan and identified 8004 insertions, 27 inversions, 6389 deletions, and 32 intrachromosomal translocations, and it also showed that long‐read sequencing identified more SVs^293^ than short‐read sequencing.

### 5.2 Clinical research on cancer

In the era of precision oncology, the development of molecular target drugs allows for the identification of specific tumor types based on genomic changes and the administration of matching therapies.^305^ Comprehensive assessment of a tumor patient's genome through sequencing can guide clinical treatment based on specific changes and help evaluate and predict cancer progression and prognosis.^306^ Several studies have used nanopore sequencing to assess genomic changes in patients with hematological malignancies.^26^, ^27^, ^307^ One study verified the feasibility of using nanopore sequencing to detect P53 gene mutations in chronic lymphocytic leukemia (CLL) patients and found that its accuracy reached 95–96% after correction by computer methods.^27^ Notably, nanopore sequencing also identified a mutation not detected by Sanger sequencing.^27^ Another study proposed a rapid mutation detection method for six acute myeloid leukemia (AML) hotspot genes based on nanopore sequencing.^26^ After testing and validating using Sanger sequencing on 22 AML patients, one study demonstrated that nanopore sequencing results for the mutation status of hotspot genes were reliable and reproducible.^26^

Clinical tumor biopsy, particularly liquid biopsy, is another promising application of nanopore sequencing.^308^ Circulating cell‐free DNA (cfDNA) in blood carries informative features of its original tissue origin^298^ and analyzing cfDNA in plasma is an effective and noninvasive strategy for obtaining tumor genomic information.^309^ Nanopore sequencing can overcome the inconvenience and high equipment cost of NGS technology for clinical cfDNA detection. One study used nanopore sequencing to detect copy number variations in cfDNA in the plasma of lung cancer patients and healthy individuals, with results highly consistent with Illumina sequencing.^309^ In terms of efficiency, nanopore sequencing can generate 2M reads within 3 h and complete the entire process, including analysis, in one working day.^309^ It can also directly detect cfDNA methylation, a marker for early cancer screening.^310^ Direct methylation detection by nanopore sequencing saves cost and time for sample processing before clinical testing while reducing sample degradation.^298^ In addition to detecting cfDNA in blood, Bruzek et al.^311^ used nanopore sequencing to detect cfDNA in cerebrospinal fluid (CSF) to determine the therapeutic effect of Pediatric high‐grade glioma. The detection of CSF samples showed a sensitivity of 85% and a specificity of 100%.^311^ Furthermore, studies have reported the use of nanopore sequencing to detect SVs in clinical ovarian and prostate cancer tumor samples.^312^

## 6 APPLICATION OF NANOPORE SEQUENCING IN PLANT GENOMES

Since the *Arabidopsis thaliana* *(L.) Heynh* genome was first sequenced and assembled using Sanger sequencing in 2000, sequencing technology has been widely used in botany.^313^ Unlike animal genomes, plant genomes often contain a large number of repetitive sequences. For example, the wheat genome contains 90% repetitive sequences.^314^ Additionally, the genome size of plants varies greatly. *Genlisea tuberosa* has the smallest genome at 61 Mb,^315^ while the *loblolly pine* has the largest genome at 22 Gb.^315^ Short‐read sequencing technology has difficulty spanning repetitive sequences and resolving polyploids in plant genomes. However, long‐read sequencing can overcome these obstacles and provides the possibility of analyzing complex plant genome sequences^314^ (Figure 4).

Oxford nanopore sequencing, the most popular long‐read sequencing platform on the market, has been applied in the field of plant genome sequencing^28^ and has resolved a series of high‐quality genomes. High‐quality reference genomes are crucial for exploring the evolutionary history of plants and for changing plant traits through artificial selection.^314^ *Oryza sativa* is an important agricultural crop, but the lack of high‐quality reference genomes has hindered researchers' understanding of its evolutionary history.^316^ Choi et al.^316^ used nanopore sequencing to assemble the genomes of two circum‐basmati rice cultivars and found strong evidence of admixture between circum‐basmati and circum‐aus cultivars. This suggests that the genome composition of some circum‐basmati varieties can be traced back to japonica.^316^ *A. thaliana* was the first important model species to obtain a full‐length genome through sequencing. However, due to the enrichment of highly repetitive elements, its new version of the reference genome still has missing or incorrect positions such as telomeres and centromeres.^317^, ^318^ Wang and colleagues^319^ obtained an almost complete high‐quality genome of *A. thaliana* named Col‐XJTU. by combining nanopore sequencing with PacBio HiFi long‐read technology.

SVs are common in plant genomes and can directly affect plant traits such as crop yield and quality.^320^ Long‐read sequencing is known to be more effective in identifying SVs, making it important to choose the right long‐read sequencing method for detecting plant SVs. In a comparison of the performance of nanopore sequencing and Pacific Biosciences (PacBio) sequencing in detecting pear SVs, Liu and colleagues^321^ found that nanopore sequencing performed better and detected more SVs than PacBio sequencing. Another study used nanopore sequencing to identify 238,490 SVs from 100 different cultivars of wild and domesticated tomato. The researchers eventually identified hundreds of SV genes^322^ that may broadly affect quantitative trait variation. By using genome editing technology to improve the SVs and expression levels of these genes, researchers were able to enhance tomato size, quantity, taste, and other traits.^322^

RNA modifications play crucial and dynamic roles in regulating gene expression during plant embryonic development, flowering, and fruit ripening. Several studies have used nanopore sequencing to detect the most common modification in plant RNA, the m6A modification.^323^ For example, Parker et al.^324^ used nanopore sequencing to directly sequence wild‐type and mRNA methylation mutants from the model plant *A. thaliana*. This allowed them to map the complexity of mRNA processing and m6A modification in both wild‐type and mutant Arabidopsis thaliana.^324^ Another study used nanopore direct RNA sequencing to detect m6A modifications in circular RNA from Phyllostachys edulis. This enabled them to pinpoint the location of m6A modifications in circular RNAs at single‐base resolution.^325^

## 7 THE ACCURACY OF NANOPORE SEQUENCING

Despite its great potential in detecting SARS‐CoV‐2, the low read accuracy of nanopore sequencing remains a significant limitation. In this section, we provide an in‐depth explanation of the reasons for this low accuracy and potential strategies for improvement.

The high error rate of nanopore sequencing is primarily due to the low accuracy of base calling.^326^ The basic principle of nanopore sequencing involves identifying the base composition or chemical modification of nucleic acid molecules by detecting the changes in current generated as they pass through the nanopore. However, the current changes caused by different bases are not as distinct as one might expect. From first‐generation to second‐generation sequencing, we have entered the era of high‐throughput sequencing, which has reduced accuracy to some extent. With third‐generation nanopore sequencing technology, single‐molecule sequencing is achieved on the basis of high‐throughput. It is understandable that during single‐molecule sequencing, the current change caused by a single base is very weak. Additionally, it is important to note that the original current signal recognized by current base‐callers does not contain only one base but multiple bases with an uncertain number. This signal is then decoded into the most likely set of bases.^326^, ^327^ Ideally, the original current signal of each recognition unit would differ by one base, but the speed at which nucleic acid passes through the nanopore is not constant, making it difficult to determine the type or position of a single base.^326^ Finally, nanopore sequencing has difficulty identifying repetitive sequences of the same base^326^ and homopolymers^97^ that exceed the length of the nanopore reading head.

Several strategies can improve the identification accuracy of nanopore sequencing, including optimizing nanopores and motor proteins, performing multiple sequencing, and updating the base‐calling algorithm.^2^ ONT has been optimizing nanopores and motor proteins.^2^ By using biological nanopores and genetic engineering techniques, they can modify the size and charge location of the nanopore, ultimately affecting the number of bases read simultaneously.^327^ Motor proteins control the speed at which nucleic acid strands pass through the nanopore. Maintaining a stable and slow passing speed helps the sensor recognize current changes and partially overcomes the problem of reading different numbers of bases at the same time.^327^ For users who urgently need high‐accuracy sequencing using nanopore sequencing, performing multiple sequencing of a single nucleic acid to generate a consensus sequence can effectively improve the quality of sequencing data.^2^ A study that conducted deep sequencing of E. coli strains with 30‐fold coverage on the MinION platform increased accuracy from 80 to 99.5%.^2^, ^328^ Additionally, developing new base‐calling algorithms is an effective way to improve the accuracy of nanopore sequencing. From past Markov model base‐calling algorithms to new‐generation neural network algorithms, the accuracy of nanopore sequencing combined with machine learning algorithms has increased to 98.3%.^329^

## 8 CONCLUSIONS AND FUTURE PERSPECTIVES

Nanopore sequencing technology, as a third‐generation sequencing technology, has the characteristics of real‐time sequencing, long reads, and high efficiency. Compared with RT‐PCR, targeted nanopore sequencing is more sensitive for diagnostic sequencing of SARS‐CoV‐2. Nanopore sequencing is cheaper and faster than Illumina sequencing. Nanopore can identify novel mutants of SARS‐CoV‐2 and identify SARS‐CoV‐2 VOCs in outbreak areas. By analyzing the nanopore sequencing results, the researchers were able to trace the origin of SARS‐CoV‐2 variants in the epidemic region and keep abreast of the lineage replacement of SARS‐CoV‐2 in the region. Nanopore sequencing can also be used to diagnose SARS‐CoV‐2 infection and other infections, and to distinguish SARS‐CoV‐2 infection from other infections. Nanopore sequencing is also used to prevent and control local outbreaks of viruses such as MPXV and NoV, particularly in developing countries with limited medical resources. It is also used for sequencing the genomes of viruses that infect plants or animals and for detecting the diversity of microbial communities.

In addition, nanopore sequencing can perform long single molecule direct RNA sequencing and obtain RNA transcriptome and epitranscriptome, which is helpful for related research on the molecular mechanism of SARS‐CoV‐2 pathogenesis, as well as the occurrence and development of cancer or the acquisition of high‐quality genomes of plant genomes. mRNA vaccines are a type of vaccine for SARS‐CoV‐2.^330^ The safety of mRNA vaccines is a concern^330^ due to the occasional adverse reactions of unknown mechanisms with the use of mRNA vaccines.^331^ Nanopore sequencing can carry out quality inspection of mRNA vaccines and ensure the safety of vaccines to a certain extent.

This article reviews the extensive application of nanopore sequencing technology in the COVID‐19 epidemic, and systematically summarizes the improvement of the ONT library preparation process and the relevant bioinformatics analysis of the mutation evolution of the SARS‐CoV‐2 virus. The use of nanopore sequencing in detecting other microbial pathogens, as well as its application in cancer and plant genomics, is also summarized. The rapid and convenient nature of nanopore sequencing is ideal for identifying regional SARS‐CoV‐2 VOCs and detecting the dynamic evolution of SARS‐CoV‐2 variants. Today, the global COVID‐19 outbreak has been effectively controlled, but regional outbreaks are still frequent. The application of nanopore sequencing will help to rapidly trace the origin of the COVID‐19 epidemic in the region and the extent of the epidemic in limited areas. At the same time, looking at the world's epidemic prevention and control forms, the highly contagious respiratory diseases caused by coronaviruses such as SARS and MERS have not disappeared. We can imagine that nanopore sequencing will play a huge role in this and the next global pandemic.

## AUTHOR CONTRIBUTION

P. Z. and S. D. contributed to the conception, design, and final approval of the submitted version. Literature was collected and analyzed by P. Z. and S. D. P. Z., Y. D., C. Z., L. L., and S. D. contributed to the manuscript writing. P. Z., Y. D., C. Z., B. L., F. Z., and S. D. contributed to the graphic design, and all authors conceived and approved the final manuscript.

## CONFLICT OF INTEREST STATEMENT

The authors declare that the study was conducted in the absence of any business or financial relationship that could be interpreted as a potential conflict of interest.

### ETHICS STATEMENT

Not applicable.

## Data Availability

Data sharing is not applicable to this article as no new data were created or analyzed in this study.
